# Risk for bipolar spectrum disorders associated with positive urgency and orbitofrontal cortical grey matter volume

**DOI:** 10.1016/j.nicl.2022.103225

**Published:** 2022-10-12

**Authors:** Ann L. Carroll, Katherine S.F. Damme, Lauren B. Alloy, Corinne P. Bart, Tommy H. Ng, Madison K. Titone, Jason Chein, Anna C. Cichocki, Casey C. Armstrong, Robin Nusslock

**Affiliations:** a Department of Psychology, Northwestern University, Evanston IL, United States; b Institute for Innovation in Developmental Sciences, Chicago IL, United States; c Department of Psychology and Neuroscience, Temple University, Philadelphia PA, United States; d Institute for Policy Research, Northwestern University, Evanston IL, United States

**Keywords:** Bipolar spectrum disorder (BSD), Positive urgency, Grey matter volume, Orbitofrontal cortex (OFC)

## Abstract

Bipolar spectrum disorders (BSDs) are associated with reward hypersensitivity, impulsivity, and structural abnormalities within the brain’s reward system. Using a behavioral high-risk study design based on reward sensitivity, this paper had two primary objectives: 1) investigate whether elevated positive urgency, the tendency to act rashly when experiencing extreme positive affect, is a risk for or correlate of BSDs, and 2) examine the nature of the relationship between positive urgency and grey matter volume in fronto-striatal reward regions, among individuals at differential risk for BSD. Young adults (ages 18–28) screened to be moderately reward sensitive (MReward; N = 42), highly reward sensitive (HReward; N = 48), or highly reward sensitive with a lifetime BSD (HReward + BSD; N = 32) completed a structural MRI scan and the positive urgency subscale of the UPPS-P scale. Positive urgency scores varied with BSD risk (MReward < HReward < HReward + BSD; *p*s≤0.05), and positive urgency interacted with BSD risk group in predicting lateral OFC volume (*p* <.001). Specifically, the MReward group showed a negative relationship between positive urgency and lateral OFC volume. By contrast, there was no relationship between positive urgency and lateral OFC grey matter volume among the HReward and HReward + BSD groups. The results suggest that heightened trait positive urgency is a pre-existing vulnerability for BSD that worsens with illness onset, and there is a distinct relationship between positive urgency and lateral OFC volume among individuals at high versus low risk for BSD. These findings have implications for understanding the expression and development of impulsivity in BSDs.

## Highlights

• Employed a high-risk design to investigate bipolar spectrum disorders (BSD).
• Elevated trait positive urgency was found to be a risk factor for developing BSDs.
• Relationship between lateral OFC volume and positive urgency differed by BSD risk.
• Findings have mechanistic implications for development of impulsivity in BSDs.

## 1 Introduction

Bipolar Spectrum Disorders (BSDs; bipolar I, bipolar II, cyclothymia) have a lifetime prevalence rate of 2.9 % and 4.4 % among adolescents and adults, respectively (Merikangas et al., 2010, National Comorbidity Survey (NSC), 2007), and are associated with short-sighted and impulsive decision-making (Moeller et al., 2001, Peluso et al., 2007, Strakowski et al., 2010, Swann et al., 2003). Elevated impulsivity predicts the onset of BSD and the occurrence of bipolar mood episodes (Alloy et al., 2009, Kwapil et al., 2000, Ng et al., 2016), and is linked with a more severe illness course (Swann et al., 2009). Perhaps, most importantly, impulsivity gives rise to some of the most damaging and costly behaviors associated with BSDs, such as behavioral addictions (Di Nicola et al., 2010), substance abuse (Alloy et al., 2009, Swann et al., 2009), and suicidality (Swann et al., 2009, Swann et al., 2005). The UPPS-P model (Cyders et al., 2007, Lynam et al., 2006) of impulsivity identifies five separable dimensions (sensation seeking, lack of premeditation, lack of perseverance, and positive and negative urgency), and of these dimensions, positive urgency, the tendency to act rashly when experiencing extremely positive emotions, is particularly implicated in BSD (Johnson et al., 2017, Johnson et al., 2016, Muhtadie et al., 2014, Victor et al., 2011).

To date, BSD research related to positive urgency has primarily focused on the severest form of the disorder, bipolar I. Further, trait positive urgency has yet to be studied within a mechanistic framework of risk for BSD, such as the Reward Hypersensitivity Model of Bipolar Disorder (described in 1.2). Thus, the objective of this study was twofold: 1) using the Reward Hypersensitivity Model of Bipolar Disorder, we employ a behavioral high-risk design to assess whether positive urgency is either a pre-existent vulnerability for or consequence of BSDs, and 2) we examine the nature of the relationship between positive urgency and reward-related brain structure among individuals at varying levels of BSD risk.

### 1.1 BSD and positive urgency

Expansive and persistent positive feelings uniquely characterize hypomania and mania (American Psychiatric Association, 2013), and are proposed to contribute to the expression of impulsive behaviors associated with BSDs (Gruber, 2011). In line with this view, elevated risk of mania, as assessed by the Hypomanic Personality Scale, is associated with heightened trait positive urgency (Giovanelli et al., 2013, Johnson et al., 2013). Individuals with bipolar I disorder who are in remission have elevated positive urgency scores compared to controls (Muhtadie et al., 2014), and, among several metrics of impulsivity, scores of positive urgency yield the largest group difference between individuals with and without bipolar I disorder (Muhtadie et al., 2014). Notably, the combination of high trait positive urgency and bipolar I disorder is linked with poorer psychosocial functioning (Muhtadie et al., 2014), lower quality of life (Victor et al., 2011), anger and aggression (Johnson & Carver, 2016), and self-harm and suicidality (Johnson et al., 2017).

Still, there is an important gap in the existing literature; little research has examined the relationship between positive urgency and milder variants of BSD (i.e., bipolar II, cyclothymia, bipolar disorder not otherwise specified). Just as with bipolar I disorder, milder BSDs can have impactful effects on an individual’s life. Subsyndromal symptoms of BSDs are associated with significant impairment and suicide risk (Altshuler et al., 2006, Kochman et al., 2005, Nusslock et al., 2008), and milder forms of BSDs often progress to more severe variants of the disorder over time (Alloy et al., 2012b, Birmaher et al., 2009, Kochman et al., 2005). Furthermore, the same mechanisms contributing to the onset of bipolar I disorder are involved in milder BSDs (Alloy et al., 2015, Nusslock and Alloy, 2017). Thus, it is important to investigate vulnerability profiles across the bipolar spectrum. The present study addresses this gap.

### 1.2 BSD and reward Hypersensitivity

The Reward Hypersensitivity Model of Bipolar Disorder (Alloy and Abramson, 2010, Alloy and Nusslock, 2019, Johnson, 2005, Nusslock and Alloy, 2017, Urosević et al., 2008) proposes that a mechanism of risk for bipolar symptoms is a hypersensitivity to reward-relevant stimuli. This reward hypersensitivity can lead to excessive approach motivation in response to reward-activating events involving goal-striving or attainment, which, in the extreme, is reflected in hypo/manic symptoms. Hypersensitivity to rewards also can lead to excessive state decreases in approach-related affect and behavior in response to reward-deactivating events involving definite failures and losses, reflected in bipolar depression symptoms. In accordance with this view, longitudinal research finds that reward sensitivity predicts increased likelihood of having a lifetime BSD (Alloy et al., 2006), increased likelihood and shorter time to onset of first lifetime BSD (Alloy, Bender, et al., 2012), recurrence of hypo/manic episodes (Alloy et al., 2008), and increased likelihood of progressing from a milder to a more severe BSD over time (e.g., bipolar II to bipolar I; Alloy et al., 2012b). Further, reward hypersensitivity is associated with hypo/manic symptoms in response to reward-striving (Nusslock et al., 2007) and reward-attainment (Johnson et al., 2000). Preliminary evidence suggests that as reward sensitivity increases, positive urgency does as well (Carlson et al., 2013).

### 1.3 BSD and Fronto-striatal structure

In line with the Reward Hypersensitivity Model, BSDs are associated with structural alterations in brain regions of a fronto-striatal reward circuit, involving the orbitofrontal cortex (OFC) and the nucleus accumbens (NAcc). The OFC is integral to regulatory and decision-making processes related to reward and emotion (Haber and Knutson, 2010, Rolls and Grabenhorst, 2008, Wallis, 2007), and can be divided into medial and lateral regions. Lateral OFC appears to be more sensitive to loss of reward (Rolls, 2019, Xie et al., 2020) and is involved in valuation of decision options (Noonan et al., 2017, Rushworth et al., 2011), whereas medial OFC is shown to be more sensitive to the presence of rewards (Grabenhorst and Rolls, 2011, Rolls, 2019) and guides value-based comparison of decision options (Noonan et al., 2017, Rushworth et al., 2011). The NAcc is a region of the ventral portion of the striatum, and primarily is involved in reward anticipation and detection (Haber and Knutson, 2010, Knutson et al., 2001, Oldham et al., 2018, Schultz, 2002).

Compared to healthy adults, individuals with a BSD exhibit bilateral reductions in grey matter volume in both medial and lateral portions of the OFC (Abé et al., 2016, Blumberg et al., 2006, Ha et al., 2009, Stanfield et al., 2009, Wise et al., 2017). Similarly, individuals at genetic risk for BSDs, but who have not yet developed the disorder, exhibit grey matter reduction in the NAcc (McDonald et al., 2004). Collectively these studies suggest that volumetric differences in reward-related brain regions are relevant to the pathophysiology of BSDs.

### 1.4 Impulsivity and Fronto-striatal structure

In a parallel body of research, aberrant structure within the fronto-striatal circuit is linked with impulsive decision-making (Pan et al., 2021), suggesting a confluence of neural abnormalities in BSD and impulsivity. Decreased prefrontal volume, particularly in the OFC, has been associated with impulsivity in healthy individuals, as measured by self-report (Matsuo et al., 2009), informant reports (Boes et al., 2009), and behavioral tasks (Li et al., 2019). In studies of severe psychopathology other than BSDs, such as schizophrenia, high trait positive urgency is associated with altered cortical structure in medial and lateral portions of the OFC (Hoptman et al., 2014). With respect to the NAcc, although fMRI studies have linked impulsive behaviors with NAcc activity (Hahn et al., 2009, Hariri et al., 2006), the volumetric findings are less conclusive (Pan et al., 2021). Thus, further research is needed to examine the relationship between impulsivity and structural abnormalities in the NAcc.

### 1.5 The current study

This study employed a behavioral high-risk design to examine trait positive urgency and fronto-striatal grey matter volume (i.e., OFC and NAcc) among individuals at varying risk for developing BSDs. A behavioral high-risk design selects participants based on behavioral characteristics that put them at-risk for developing a specific disorder. The current study defined risk as self-reported reward sensitivity given that it predicts BSD first onset and severity (Alloy, Bender, et al., 2012; Alloy et al., 2012b). As such, three groups of participants were examined: individuals at low-risk for BSD (moderate-reward sensitivity and no BSD diagnosis; MReward), high-risk without a BSD (high reward sensitivity and no BSD diagnosis; HReward), and high-risk with a BSD (high reward sensitivity and a BSD diagnosis; HReward + BSD). The behavioral high-risk approach allowed us to distinguish whether trait positive urgency and fronto-striatal volume alterations reflect a pre-existing risk factor for BSD, or a correlate of the illness. The participants in the study sample were 18–28 years old, which represents a critical period of brain development in the prefrontal cortex (Ostby et al., 2009) and BSD onset (Weissman et al., 1996).

For all analyses, we expected to find that the HReward and HReward + BSD groups would display similar profiles across different variables of interest (i.e., positive urgency, grey matter volume) as compared to MReward individuals. Results in line with this prediction would suggest that both elevated positive urgency and fronto-striatal structural profiles predate the onset of a BSD diagnosis and may reflect a preexisting risk profile. Accordingly, we made the following hypotheses: 1) we predicted individuals in both the HReward and HReward + BSD groups would display elevated positive urgency scores relative to the MReward group. 2) We predicted that across all BSD risk groups, we would observe an association between elevated positive urgency scores and reduced OFC volumes, and that this relationship would be amplified in the HReward and HReward + BSD groups. 3) We predicted that reduced NAcc grey matter volume would be associated with elevated positive urgency scores across all BSD risk groups. As with the OFC, we anticipated the relationship between NAcc volume and positive urgency would be amplified in HReward and HReward + BSD individuals.

## 2 Methods

### 2.1 High-risk design

Participants were recruited from the Teen Emotion and Motivation (TEAM) Project, a large, ongoing longitudinal project in the Philadelphia area that prospectively identified individuals at risk for a BSD based on self-reported reward sensitivity (see Supplemental Fig. 1 for overview of Project TEAM timeline). Project TEAM used a two-stage recruitment procedure (Alloy et al., 2012a). During Stage 1 screening, 9,991 students (ages 14–19) completed two measures: The Behavioral Inhibition System/Behavioral Activation System scales (BIS/BAS; Carver & White, 1994) and the Sensitivity to Punishment/Sensitivity to Reward Questionnaire (SPSRQ; Torrubia, Ávila, Moltó, & Caseras, 2001). Participants scoring in the 40th to 60th percentile on both the Total BAS subscale of the BIS/BAS scales and the Sensitivity to Reward subscale of the SPSRQ were classified as having moderate reward sensitivity and considered at low-risk for BSD (n = 750). Participants scoring in the 85th to 100th percentile on both measures were classified as having high reward sensitivity and considered at high-risk for BSD (n = 1,200). Participants low in reward sensitivity were not included in the study due to budgetary restrictions, and because reward hyposensitivity is a better predictor of depression than hypo/mania (Alloy et al., 2016). From this initial Stage 1 screening, 539 individuals (334 high reward and 205 moderate reward) returned for Stage 2 screening, during which participants were administered a diagnostic interview using the expanded Schedule for Affective Disorders and Schizophrenia - Lifetime interview (exp-SADS-L; Alloy, Bender, et al., 2012; Endicott & Spitzer, 1978). Based on the exp-SADS-L, participants with a lifetime BSD or psychosis spectrum disorder at Stage 2 screening were excluded from Project TEAM in order to prospectively assess risk for the onset of BSDs.

At the beginning of the longitudinal portion of Project TEAM (i.e., following Stages 1 and 2), there were 497 participants enrolled (307 high-reward sensitive and 190 moderate-reward sensitive). Participants completed biannual, in-person follow-up sessions that included a diagnostic interview using the expanded SADS-Change interview (exp-SADS-C; Endicott and Spitzer, 1978), and self-reported measures (e.g., UPPS-P, detailed in 2.3). The exp-SADS-C was administered to detect new diagnoses, particularly conversion to or worsening of BSDs (i.e., bipolar I, bipolar II, cyclothymia, and bipolar not otherwise specified). We report in a prior publication that the rate of BSD onset in the HReward group was 12.9 % within an average of 12.8 months of follow-up (Alloy et al., 2012a), and increased to 15 % by an average of 31.7 months. This allowed us to form a new BSD group, which became the foundation for the current project. The rate of participant conversion to BSDs in Project TEAM was consistent with prior epidemiological data (Alloy et al., 2012a, Birmaher et al., 2009, Van Meter et al., 2021). The study was approved by the Temple University Institutional Review Board. Prior to participation in Project TEAM, participants aged 14–17 years provided informed written assent as well as informed written parental consent; participants aged 18 or 19 years provided informed written consent.

### 2.2 Participants

Participants in this study were recruited from Project TEAM to do a follow-up, single MRI session. Information from Stage 1, Stage 2, and follow-up sessions determined eligibility for recruitment into the single session MRI scan. Specifically, BIS/BAS and SPSRQ scores at Stage 1 of Project TEAM determined reward sensitivity groupings (i.e., MReward, HReward), and diagnostic information from the participant’s most recent exp-SADS-C prior to the MRI session determined the presence/absence of a BSD (e.g., HReward + BSD). The mean time between the exp-SADS-L and MRI session for participants was approximately 2 years (mean = 2.02 years, standard deviation = 2.27), which provided a sufficient time window for BSD conversion. The absence/presence of BSD diagnoses was confirmed on the day of the scan.

A total of 130 young adults completed the MRI scan. We excluded participants from the MRI session based on the following criteria: ferrous metal in any part of the body, lifetime history of head trauma, claustrophobia, left-handedness, and pregnancy. Eight additional participants were excluded from analyses in the current study because they did not complete the UPPS-P questionnaire. Thus, the final analytic sample for this study included 122 young adults (51 % female; mean age at scan = 20.98; age range 18–27): 42 MReward (low-risk) individuals, 48 HReward (high-risk) individuals, and 32 HReward + BSD individuals (3 bipolar I, 18 bipolar II, 3 cyclothymia, and 8 bipolar not otherwise specified). Although the MRI sample is cross-sectional, it benefits from the longitudinal design of Project TEAM, as all participants in the MRI sample with a BSD converted from high-risk to illness after initial prospective screening. The sample was 57.0 % Caucasian, 23.1 % African American, 9.9 % Asian or Pacific Islander, 6.6 % Biracial/Multiracial, 0.8 % Native American and 3.3 % Other race participants. Additionally, 5.8 % of participants identified as Hispanic/Latino. Participants provided informed written consent, given that all were over 18 years of age at the time of the scan, and the IRB at Temple University approved all study protocols.

### 2.3 Self-Report measures

To assess self-reported reward sensitivity at the initial Stage 1 recruitment into Project TEAM, participants completed the BIS/BAS (Carver & White, 1994) and the SPSRQ (Torrubia et al., 2001). We focused our recruitment on the BAS-Total and Sensitivity to Reward subscales from these measures. The internal consistencies for the BAS-Total and Sensitivity to Reward scales at Stage 1 screening were α’s = 0.80 and 0.76, respectively.

Expanded versions of the SADS-L and SADS-C (Alloy et al., 2008, Endicott and Spitzer, 1978) were administered at the Stage 2 recruitment into Project TEAM and follow-up interviews, respectively, by trained diagnosticians to assess for the presence versus absence of a lifetime BSDs. The exp-SADS-L has demonstrated inter-rater reliability within our lab of κ > 0.80 (Alloy et al., 2008).

At follow-up sessions, participants completed the Positive Urgency (PU) scale of the UPPS-P self-report questionnaire (Lynam et al., 2006), which is a 14-item Likert-type scale that measures the dispositional tendency to act rashly when experiencing extreme positive emotion. An example item is, “I tend to lose control when I am in a good mood.” The current study analyzed UPPS-P data from the follow-up session closest to the MRI session date (i.e., either preceding or following the MRI session). Not all participants completed every follow-up session or UPPS-P administration. Thus, some participants’ UPPS-P completion dates were closer in time to their MRI session than others. The average time between the UPPS-P administration and the MRI scan was 198.34 days (standard deviation = 238.66 days). See Table 1 for the median and mean number of days between UPPS-P administration and MRI by BSD risk group. Internal consistency in this sample was α = 0.92. Items were recoded so that higher mean scores on the PU scale represent higher levels of impulsivity.

### 2.4 MRI acquisition and analysis

Imaging data were collected at Temple University using a Siemens 3 T Verio scanner with a 12-channel head coil. Structural MPRAGE images were collected using the following parameters: repetition time (TR) = 1600 ms, echo time (TE) = 2.46 ms, field-of-view (FOV) = 252, flip angle = 9, voxel size = 0.5 × 0.5 × 1.0 mm, number of interleaved slices = 176.

FreeSurfer version 6.0 automatic segmentation software extracted grey matter volume estimates (http://surfer.nmr.mgh.harvard.edu/; Fischl et al., 2012). MRI data were visually inspected for quality of segmentation and parcellation consistent with the visual description in Raamana et al., 2021; multiple individual raters examined each scan, any errors were reviewed for manual edit by KSFD and were completed only if the error was verifiable in two planes of visualization (n = 23; 17.6%). There were no significant outliers in the volume data for any region-of-interest (ROI). A priori ROIs were restricted to regions integral to the frontal-striatal circuit in order to limit multiple comparisons: bilateral OFC and bilateral NAcc. We examined both the lateral OFC as well as medial OFC. Individual surfaces were averaged using a non-rigid, high-dimensional spherical method that relies on the alignment of cortical folding patterns. OFC and NAcc volumes were extracted using the Desikan-Killiany atlas (Desikan et al., 2006).

### 2.5 Statistical analyses

For all analyses, Fisher’s protected t-tests (Cohen et al., 2003) were employed to minimize familywise error rate, which requires a significant omnibus ANOVA F-test in order to proceed to pairwise comparisons. To test the relationship between BSD risk group and positive urgency scores, we conducted a one-way Analysis of Covariance (ANCOVA), controlling for psychotropic medication status (on versus off) and date of UPPS-P administration. We also controlled for sex and age at scan, given previous research demonstrating their role in the expression of impulsivity (Cross et al., 2011, Steinberg et al., 2008). To test the association between BSD risk group, positive urgency, and fronto-striatal grey matter volume, we conducted a Group (MReward, HReward, and HReward + BSD) × positive urgency ANCOVAs on each of the grey matter volume ROIs, separately (lateral OFC, medial OFC, NAcc), controlling for total brain volume (total gray matter, white matter, and ventricles; FreeSurfer “BrainSeg”), psychotropic medication, and date of UPPS-P administration. We again controlled for sex and age at scan, due to their established relationship with brain volume (Cosgrove et al., 2007, Kennedy et al., 2009).

## 3 Results

We examined the BSD risk groups (MReward, HReward, HReward + BSD) on demographic variables using analyses of variance and chi-square tests. There were no significant between-group differences in age (*F*(2, 119) = 0.16, *p* =.85, partial η^2^ < 0.01), sex, (χ^2^(2) = 0.05, *p* =.98), medication at MRI scan (χ^2^(2) = 0.89, *p* =.64), race (χ^2^(10) = 11.49, *p* =.32), or time between the MRI scan and UPPS-P administration (*F*(2, 119) = 0.12, *p* =.89, partial η^2^ < 0.01); see Table 1.

**Table 1:** Sample Characteristics by BSD Risk Group.

	MReward (n = 42)	HReward (n = 48)	HReward + BSD n = 32)
Age at scan (years; mean ± sd)	21.12 ± 1.92	20.90 ± 1.94	20.91 ± 2.28
Female (%)	50	50	53.13
Psychotropic medication at scan (#)	5	6	2
SSRI	4	3	0
SNRI	0	1	0
NDRI	0	0	1
Benzodiazepine	0	1	0
Mood stabilizer and antipsychotic	0	0	1
Other	1	1	0
Race/ethnicity (%)			
Asian	7.14	8.33	15.63
Black	28.57	27.08	9.38
Native American	0	0	3.13
White	59.52	52.08	59.38
Bi-/Multiracial	2.38	10.42	6.25
Other/Unknown	2.38	2.08	6.25
Time between MRI and UPPS-P (|days|)			
Median	104.5	83.0	75.5
Mean ± sd	190.26 ± 205.77	211.75 ± 258.14	189.00 ± 258.21

Note. BSD = bipolar spectrum disorder, MReward = moderate reward sensitivity, HReward = high reward sensitivity, HReward + BSD = high reward sensitivity with a bipolar spectrum disorder, |days|=absolute value of days between the MRI scan UPPS-P administration, sd = standard deviation.

### 3.1 Positive urgency analyses

**Fig. 1:**
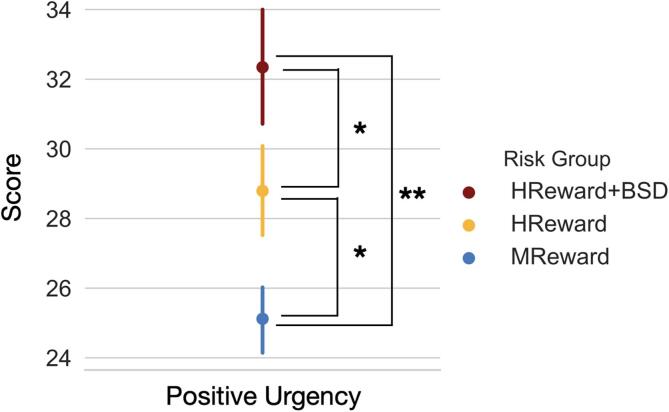
Mean positive urgency scores by bipolar spectrum disorder risk group, with confidence intervals of 68 %. Note: *p≤0.05, **p <.01; MReward = moderate reward sensitivity, HReward = high reward sensitivity, HReward + BSD = high reward sensitivity with a bipolar spectrum disorder.

There was a main effect of BSD risk group on positive urgency scores (*F*(2, 115) = 6.92, *p* =.001, partial η^2^ = 0.11; Fig. 1), controlling for sex, age at scan, medication status, and date of UPPS-P administration. Consistent with our first hypothesis, follow-up analyses indicated that individuals in the MReward group (M = 25.12, SD = 6.21) reported significantly lower trait positive urgency scores than both the HReward (M = 28.79, SD = 9.15; *t*(1, 115) = -2.0, *p* =.05) and HReward + BSD groups (M = 32.34, SD = 9.47; *t*(1, 115) = -3.71, *p* <.001). Participants in the HReward group also reported lower trait positive urgency than the HReward + BSD group (*t*(1, 115) = -1.97, *p* =.05). There was a significant relationship between positive urgency and age at scan (*F*(1, 115) = 4.30, *p* =.04, partial η^2^ = 0.04), such that positive urgency decreased with age (b = -0.78). There was no relationship between positive urgency scores and either sex (*F*(1, 115) = 1.62, *p* = 0.21, partial η^2^ = 0.01), medication (*F*(1, 115) = 0.38, *p* =.54, partial η^2^ < 0.01), or date of UPPS-P administration (*F*(1, 115) = 0.03, *p* =.86, partial η^2^ < 0.001).

### 3.2 Positive urgency and ROI volume analyses

#### 3.2.1 Lateral orbitofrontal cortex

There was a main effect of positive urgency on lateral OFC volume, (*F*(1,111) = 12.53, *p* <.001, partial η^2^ = 0.10) controlling for sex, age at scan, medication status, date of UPPS-P administration, and total brain volume, such that heightened positive urgency was associated with a smaller lateral OFC volume (b = -101.40). There also was a main effect of BSD risk group on lateral OFC volume, (*F*(2,111) = 6.84, *p* =.002, partial η^2^ = 0.11), such that lateral OFC volume was larger among participants at heightened risk for developing a BSD, and among participants with a BSD than those at low risk for BSD (MReward M = 15160.07, SD = 1966.29; HReward M = 15436.67, SD = 2026.30; HReward + BSD M = 15807.59, SD = 2318.931). Follow-up analyses indicated, however, that although the omnibus F was significant, none of the BSD risk groups significantly differed from each other on lateral OFC volume, (MReward versus HReward: *t*(1, 111) = -1.61, *p* =.11); MReward versus HReward + BSD: (*t*(1, 111) = -1.49, *p* =.14); HReward versus HReward + BSD: (*t*(1, 111) = -0.11, *p* =.91)). There was no relationship between lateral OFC volume and either sex (*F*(1, 111) = 0.44, *p* =.51, partial η^2^ < 0.01), age at scan (*F*(1, 111) = 0.39, *p* =.53, partial η^2^ < 0.01), medication status (*F*(1, 111) = 1.36, *p* = 0.26, partial η^2^ = 0.01), or date of UPPS-P administration (*F*(1, 111) = 0.07, *p* =.79, partial η^2^ < 0.001).

**Fig. 2:**
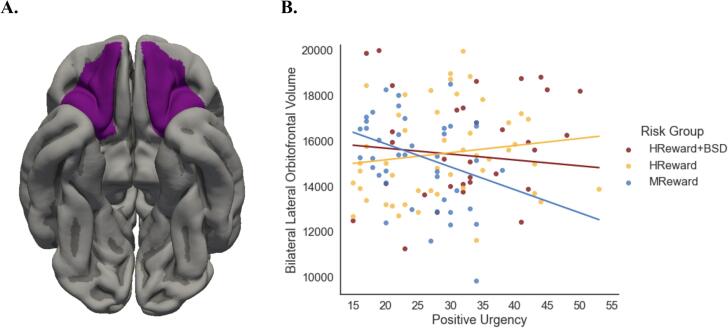
**A.** The lateral OFC ROI. **B.** The simple slopes of each BSD risk group and the interaction between Positive Urgency × BSD risk group in predicting bilateral lateral OFC volume. Note: OFC = orbitofrontal cortex, ROI = region-of-interest, MReward = moderate reward sensitivity, HReward = high reward sensitivity, HReward + BSD = high reward sensitivity with a bipolar spectrum disorder.

Regarding our second hypothesis, these main effects were qualified by a significant interaction between BSD risk group and positive urgency (*F*(2,111) = 8.30, *p* <.001, partial η^2^ = 0.13), suggesting that the relationship between trait positive urgency and lateral OFC volume was contingent on one’s risk status for developing a BSD (Fig. 2). Investigating the simple regression slopes indicated that, in line with the hypothesis, lateral OFC volumes decreased as positive urgency scores increased in MReward individuals (b = -101.41, *SE* = 28.65, *t* = -3.54, *p* <.001). However, contrary to prediction, the HReward (b = 31.49, *SE* = 18.44, *t* = 1.71, *p* =.09) and HReward + BSD groups (b = -26.01, *SE* = 21.83, *t* = -1.14, *p* =.24) did not show the expected association between positive urgency and bilateral OFC volume. The differences in slopes were significantly different between the MReward and HReward groups (b = -132.90, *t*(1, 111) = -3.92, *p* <.001), as well as the MReward and HReward + BSD groups (b = -75.40, *t*(1, 111) = -2.09, *p* =.04). The HReward group had a slightly more positive slope than the HReward + BSD group (b = 57.5, *t*(1, 111) = 2.03, *p* =.04), however, we did not interpret this finding because the simple slopes analysis indicated that neither group’s slope differed from zero.

#### 3.2.2 Medial orbitofrontal cortex

Contrary to our second hypothesis, neither positive urgency (*F*(1,111) = 1.44, *p* =.23, partial η^2^ = 0.01), BSD risk group (*F*(2,111) = 1.40, *p* =.25, partial η^2^ = 0.02), nor their interaction (*F*(2,111) = 1.65, *p* =.20, partial η^2^ = 0.03) were associated with medial OFC volume.

#### 3.2.3 Nucleus accumbens

Contrary to our third hypothesis, neither positive urgency (*F*(1,111) = 2.06, *p* =.15, partial η^2^ = 0.02), BSD risk group (*F*(2,111) = 1.30, *p* =.28, partial η^2^ = 0.02), nor their interaction (*F*(2,111) = 1.74, *p* =.18, partial η^2^ = 0.03) were associated with NAcc volume.

## 4 Discussion

This study employed a mechanistically-based behavioral high-risk design to 1) investigate the association between positive urgency and risk for BSD, and 2) examine the relationship between positive urgency and fronto-striatal grey matter volume among individuals at differential risk for BSD. Self-reported reward sensitivity was used to establish profiles of risk for the initial recruitment into Project TEAM. Our use of a behavioral high-risk design allowed us to test whether relationships with positive urgency predate the onset of BSDs and reflect a possible preexistent risk factor for the illness or reflect a correlate of illness.

Consistent with our first hypothesis, individuals with a BSD (HReward + BSD group) had higher positive urgency scores than a low-risk comparison group without a BSD (MReward group). This is a replication and extension of previous research showing that trait impulsivity, and positive urgency, in particular, is elevated in individuals with mild as well as severe BSDs, as compared to those without. Also extending prior work, we demonstrate that individuals at-risk for BSDs, based on the Reward Hypersensitivity Model of Bipolar Disorder, but who have not yet developed the illness (i.e. HReward group), reported higher positive urgency scores than participants in the low-risk MReward comparison group. This suggests that heightened positive urgency predates the onset of BSDs. In regards to our second prediction, lateral OFC volume decreased as positive urgency increased among individuals in the MReward group. This finding is consistent with previous research linking impulsive decision-making with reduced prefrontal grey matter volume (Bjork et al., 2009, Boes et al., 2009, Matsuo et al., 2009). Contrary to prediction, however, this association was not observed among either the HReward or HReward + BSD groups. Rather, the individuals in these two groups showed little to no association between positive urgency and lateral OFC volume. Medial OFC and NAcc grey matter volumes were not found to be related to positive urgency scores or BSD risk group.

Impulsivity is a core feature of BSDs (American Psychiatric Association, 2013, Moeller et al., 2001, Peluso et al., 2007, Strakowski et al., 2010, Swann et al., 2003), and underlies damaging and costly behaviors exhibited in BSD illness, such as substance abuse (Alloy et al., 2009, Swann et al., 2009), compulsive gambling and buying (Di Nicola et al., 2010), and suicidality (Swann et al., 2009, Swann et al., 2005). Positive urgency is a dimension of impulsivity suggested to be particularly relevant to the presence and severity of bipolar I disorder (Giovanelli et al., 2013, Johnson et al., 2017, Johnson et al., 2016, Johnson et al., 2013, Muhtadie et al., 2014, Victor et al., 2011). To build upon the existing literature on bipolar I disorder, the present study included milder variants of the disorder (i.e., bipolar II, cyclothymia, bipolar not otherwise specified), as well as individuals at elevated risk for BSDs, as determined by the Reward Hypersensitivity Model of Bipolar Disorder. The inclusion of milder variants was important; to date, many BSD studies singularly focus on bipolar I disorder, as it is the most severe form of BSD illness. However, there is strong evidence that milder BSD variants also are associated with significant impairment and suicidality (Altshuler et al., 2006, Kochman et al., 2005, Nusslock et al., 2008), can, crucially, progress to more severe illness over time (Alloy et al., 2012b, Birmaher et al., 2009, Kochman et al., 2005), and involve the same underlying mechanisms that contribute to bipolar I onset (Alloy et al., 2015, Nusslock and Alloy, 2017). Thus, investigating the vulnerability profile of milder BSDs, such as with positive urgency tendencies, has important implications for understanding mechanisms involved in the entire bipolar spectrum, including bipolar I disorder. We report, for the first time, that heightened positive urgency may be a precursor to BSD onset, and possibly signifies a risk factor for developing the illness among those that are particularly sensitive to rewards. That is, individuals with the high-risk profile (i.e., HReward and HReward + BSD), regardless of whether or not they had a BSD, share a tendency to act more impulsively when experiencing extremely positive emotion. This is in contrast to MReward individuals, who reported fewer impulsive behaviors when experiencing extremely positive emotion. Replicating prior work (Muhtadie et al., 2014), we also found that, among all groups, individuals with a BSD displayed the highest levels of positive urgency. Collectively, this suggests that high positive urgency may be a preexistent risk factor for BSDs, not merely a consequence of the illness, and may worsen with illness onset.

In regards to our second prediction, we also report that elevated positive urgency was associated with reduced lateral OFC grey matter volume among individuals in the MReward group. This finding is consistent with previous research suggesting a negative association between prefrontal grey matter volume and impulsivity (Bjork et al., 2009, Boes et al., 2009, Churchwell et al., 2010, Ersche et al., 2011, Matsuo et al., 2009). A range of regulatory processes rely on OFC input, including reward valuation (Haber & Knutson, 2010) and emotion-based decision-making (Rolls & Grabenhorst, 2008). Lateral portions of the OFC are particularly implicated in evaluating punishing information to direct future behavior (Rolls, 2019, Xie et al., 2020), and as such, we posit that among healthy individuals, lower lateral OFC grey matter volume may contribute to a diminished capacity to both evaluate negative decision options and inhibit rash behaviors.

Interestingly, there was no relationship between lateral OFC volume and positive urgency among either the HReward or HReward + BSD individuals. This was unexpected given that heightened trait impulsivity (Bjork et al., 2009, Boes et al., 2009, Churchwell et al., 2010, Ersche et al., 2011, Matsuo et al., 2009) and BSDs (Abé et al., 2016, Blumberg et al., 2006, Ha et al., 2009, Stanfield et al., 2009, Wise et al., 2017) both have been linked with reduced OFC volume. Although future research is needed to better understand this lack of association between positive urgency and OFC grey matter volume in individuals at high-risk or with a BSD, we put forth a potential explanation to help guide subsequent work. We propose that by sampling for high reward sensitivity, which is one mechanistic pathway to BSD, we selected a subgroup of individuals with, and at-risk for, BSD that display heightened reward-related brain function and structure. In line with this view, previous research has reported that high self-reported reward sensitivity is associated with increased ventral striatal activation to monetary reward cues (Caseras et al., 2013), and greater OFC volume (Nusslock et al., 2014), compared to individuals with moderate reward sensitivity. Likewise, in the present study we observed a main effect of group on lateral OFC volume, such that the HReward and HReward + BSD groups displayed a nonsignificant trend towards elevated volume compared to the MReward group. Collectively, this may suggest that by recruiting individuals with heightened reward sensitivity, we may have restricted variation in OFC volume among HReward and HReward + BSD participants, thus limiting our ability to see relationships between positive urgency and OFC volume among high-risk participants. Future studies on the relationship between positive urgency, reward-related brain structure, and BSD might consider recruiting participants based on other risk criteria (e.g., family risk status), in addition to reward sensitivity, given that different risk profiles of BSD may be associated with distinct profiles of OFC function and structure. The present study, however, provides insight into the possible role of OFC volume in trait positive urgency among individuals at differential risk for BSD, as defined by reward sensitivity.

Finally, we did not find the predicted relationships between risk for BSD, positive urgency, and grey matter volume in either the NAcc or medial OFC. There are a few possible explanations for these null results. For one, whereas reduced NAcc grey matter volumes are found in BSDs (Haller et al., 2011, Haznedar et al., 2005, Lisy et al., 2011, McDonald et al., 2004, Scherk et al., 2008), there are inconsistent NAcc volumetric findings in the impulsivity literature (Pan et al., 2021). The null finding for the NAcc in the present study combined with inconsistencies in prior research on the NAcc and impulsivity may suggest that OFC grey matter volume may be more central to positive urgency tendencies than NAcc volume. Rash actions and decisions typically are modulated by top-down prefrontal executive control (Buckholtz and Meyer-Lindenberg, 2012, Dalley et al., 2011) and, as such, prefrontal structural integrity may be more influential in the expression of positive urgency than is striatal volume. Second, the medial OFC may be less relevant to the expression of positive urgency than the lateral OFC. The medial and lateral OFC are implicated in different forms of impulsivity (Dalley and Robbins, 2017), and as such, heightened positive urgency may involve impairment of processes supported by the lateral OFC, such as valuation of choice options (Noonan et al., 2017, Rushworth et al., 2011) and/or sensitivity to negative consequences (Rolls, 2019, Xie et al., 2020).

### 4.1 Study limitations and Future directions

The present study should be considered in the context of its limitations. First, the cross-sectional nature of the data precludes any causal interpretation. A multi-wave, longitudinal study is needed to determine whether heightened positive urgency and lateral OFC grey matter volume predict BSD onset. It would be important for such a study to assess baseline measures of positive urgency, which the present study was not able to do because the UPPS-P questionnaire only was administered after Project TEAM’s initial screening stages. Such a study also could clarify the developmental trajectory of volumetric differences associated with elevated positive urgency in low and high-risk samples. For example, it is unclear at what point in neural development that an association between positive urgency and lateral OFC grey matter volume emerges, and for how long it persists. Second, it is necessary to examine the relationship between positive urgency and BSDs in relation to brain function (i.e., fMRI), in addition to structure. A couple of studies suggest that fronto-striatal function plays a role in trait positive urgency (Johnson et al., 2019, Zhao et al., 2017), and it is currently unclear whether structure or function is more central to positive urgency expression in BSDs. Third, the current study only examined grey matter volume, because it could be similarly interpreted and calculated across cortical and subcortical volumes. Future studies would benefit from a more comprehensive examination of morphometry metrics relevant for cortical structures (Abé et al., 2016, Hoptman et al., 2014) and subcortical structures (Caseras et al., 2015, Mamah et al., 2016, Quigley et al., 2015), in order to examine other features of the brain that may be relevant to positive urgency (e.g., surface area, thickness, shape). Finally, budgetary restrictions prevented the inclusion of individuals with low-reward sensitivity as a comparison group to those with moderate or high reward sensitivity. Prior research shows such a group would be vulnerable for unipolar depression or Major Depressive Disorder without a history of hypomania or mania (Alloy, Olino, Freed & Nusslock, 2016). As a result, we cannot generalize our findings across the entire spectrum of reward sensitivity or to risk for unipolar depression, and further research is needed to address these topics.

## 5 Conclusion

The present study is the first to use a mechanistic, high-risk framework to assess the relationship between positive urgency and risk for BSDs. We report heightened positive urgency among both individuals with a BSD diagnosis (i.e. HReward + BSD) and individuals at elevated risk for a BSD but who have not yet developed the illness (i.e. high reward sensitivity; HReward). Presence of a BSD was associated with the greatest elevation in positive urgency scores, and the HReward profile was intermediate between those at average risk (i.e., moderate reward sensitivity; MReward) and those with a BSD. Together, the findings suggest that heightened positive urgency may be preexistent to, and exacerbated by, a BSD onset. Future longitudinal research should test whether heightened positive urgency is a prospective risk factor for BSD onset. The present study is also the first to test the relationship between positive urgency, BSD risk, and grey matter volume of fronto-striatal regions. In line with prediction, heightened positive urgency was associated with lower bilateral lateral OFC grey matter volume among individuals at low-risk for BSDs (i.e., moderate reward sensitivity; MReward). This normative and expected negative relationship, however, was not found in individuals in the HReward or HReward + BSD groups. Rather, these two groups exhibited trend-level elevations in lateral OFC grey matter volume as compared to the MReward group, which may be a result of recruiting for and investigating a reward hypersensitivity mechanistic pathway to BSD illness. Last, we report no association between positive urgency and grey matter volume in either the medial OFC or NAcc. Based on the results, we suggest that lateral OFC volume may be a region particularly central to the expression of positive urgency tendencies. Although further research is needed to investigate the neural mechanisms underlying the reported effects, we find, for the first time, evidence of a differential relationship between lateral OFC grey matter volume and positive urgency among individuals with low-risk and high-risk (with or without a diagnosis) profiles for developing BSDs.

## Funding

This research was supported by National Institute of Mental Health (NIMH) Grant MH077908 and MH126911 to Lauren B. Alloy. Preparation of the manuscript also was supported by NIMH Grant MH100117 to Robin Nusslock and National Institute of Health (NIH) grant T32 NS047987 to Ann L. Carroll. Funding sources did not have a role in the conduct of the study or the preparation and submission of the article.

## CRediT authorship contribution statement

**Ann L. Carroll:** Conceptualization, Formal analysis, Writing – original draft. **Katherine S.F. Damme:** Formal analysis, Writing – review & editing. **Lauren B. Alloy:** Conceptualization, Funding acquisition, Methodology, Writing – review & editing. **Corinne P. Bart:** Investigation, Writing – review & editing. **Tommy H. Ng:** Investigation, Writing – review & editing. **Madison K. Titone:** Investigation, Writing – review & editing. **Jason Chein:** Supervision, Writing – review & editing. **Anna C. Cichocki:** Writing – review & editing. **Casey C. Armstrong:** Formal analysis, Writing – review & editing. **Robin Nusslock:** Conceptualization, Funding acquisition, Methodology, Writing – original draft.

## Declaration of Competing Interest

The authors declare that they have no known competing financial interests or personal relationships that could have appeared to influence the work reported in this paper.

## Data Availability

Position on/ability to share data needs to be confirmed with PIs, if article is accepted
