# Supplementary material for: Risk for bipolar spectrum disorders associated with positive urgency and orbitofrontal cortical grey matter volume
Source: Neuroimage Clin. 2022 Oct 12;36:103225. doi: 10.1016/j.nicl.2022.103225 (PMC9668630; doi:10.1016/j.nicl.2022.103225)
Supplement: Supplementary data 1 [file mmc1.docx]

**Methods Supplement**


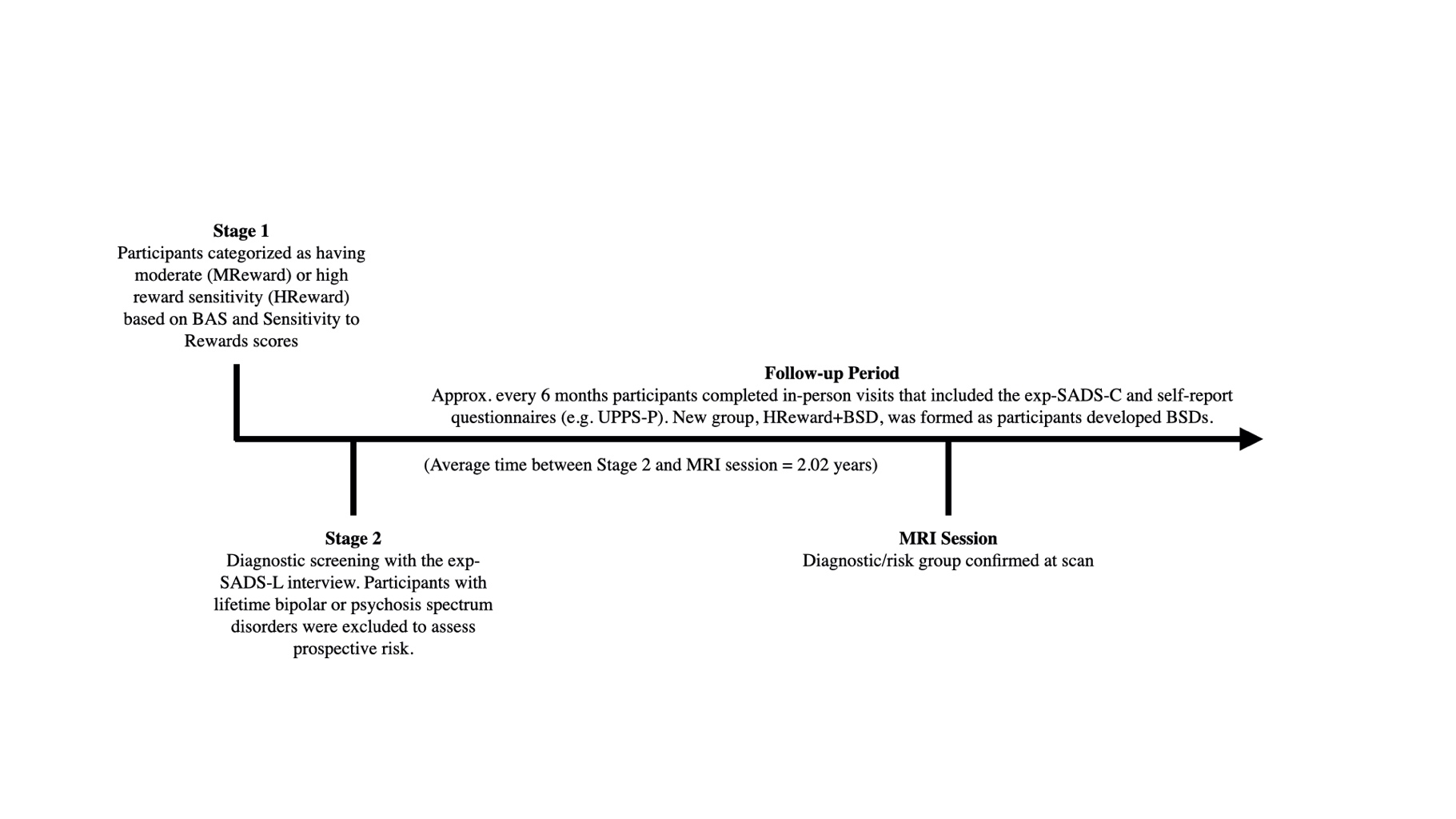


Supplement Fig. 1. The order of events and timeline of Project TEAM and current MRI study. The average time between Stage 2 screening and the MRI session was approximately 2 years, and the average time between the MRI session and the UPPS-P questionnaire was about 198 days.

Note: MReward=moderate reward sensitivity, HReward=high reward sensitivity, HReward+BSD=high reward sensitivity with a bipolar spectrum disorder, BAS=Behavioral Activation System, SADS-L= Schedule for Affective Disorders and Schizophrenia – Lifetime, SADS-C= Schedule for Affective Disorders and Schizophrenia – Change, BSD=bipolar spectrum disorder.
